# An AHP-ME-IOWA Model for Assessing National Space Technology Scientific and Technological Strength: A Case Study of the United States

**DOI:** 10.3390/e27111141

**Published:** 2025-11-06

**Authors:** Yingying Chen, Zhenqiang Qi, Jinzhao Li, Yuting Zhu

**Affiliations:** 1 China Aerospace Academy of Systems Science and Engineering, Beijing 100048, China; 2 China Academy of Launch Vehicle Technology, Beijing 100076, China

**Keywords:** space technology, analytic hierarchy process, induced ordered weighted averaging, maximum entropy

## Abstract

Space technology, a frontier of global scientific innovation, is crucial for competitive edges and national tech innovation. Amid intensified international competition and rapid technological change, scientifically evaluating a country’s Scientific and Technological Strength in Space Technology (STSST) is vital. A model is innovatively proposed in this study called “Analytic Hierarchy Process-Maximum Entropy-Induced Ordered Weighted Average (AHP-ME-IOWA)” for the assessment of STSST. First, an STSST assessment indicator system is developed with four sub-dimensions: scientific research, industrial operation, innovation output, and policy resources. Second, the AHP model is used to convert experts’ qualitative judgments on indicator importance into initial individual weight vectors. Subsequently, the IOWA operator is employed to aggregate these individual weight vectors, thereby mitigating the impact of outliers and enhancing the robustness of the weights. Specifically, the weights are reordered using the cosine similarity between each expert’s weight vector and the temporary group mean as the induced value. Position weights are then determined via the ME method, and consensus weights are derived through re-aggregation. A systematic evaluation of the United States’ STSST was conducted using this method. The results show that the United States achieved a comprehensive STSST score of 8.73 (out of 10), which is in line with the actual situation, thereby providing empirical validation for the proposed method.

## 1. Introduction

In the long journey of human exploration of the cosmos, the space endeavor has always played a pivotal role. As the forefront of global scientific innovation, space technology is key to gaining strategic advantages and serves as a powerful engine propelling national technological innovation and development [1]. With the intensification of international competition and accelerated technological changes, countries’ investment and dependence on space technology are constantly increasing. In this context, it has become particularly important to accurately measure and continuously monitor a country’s Scientific and Technological Strength in Space Technology (STSST).

In recent years, comprehensive research capacity assessments have garnered significant attention both domestically and internationally. Current research exhibits a trend toward diversification and specialization, with various institutions adopting tailored evaluation frameworks and indicator systems. These studies extensively utilize rich and rigorous quantitative analysis tools in terms of methodology, covering various methodologies such as open source data mining extraction [2], bibliometric statistics [3,4], patent depth statistical analysis [5], collaborative network tightness calculation [6], and so on. On this basis, global scientific and technological information database resources such as the Web of Science (WoS) database and the Derwent Innovations Index (DII) patent database, and a technology literature comprehensive retrieval platform are fully utilized to continuously track and accurately grasp the long-term trends and short-term dynamics of global scientific and technological development. This has provided us with a lot of inspiration for our research work.

Despite these advancements, existing methodologies present notable limitations. On one hand, some studies lean heavily toward qualitative assessments, lacking robust quantitative data support. For instance, reports such as the U.S. Government Accountability Office’s Technology Assessment Design Handbook: Handbook for Key Steps and Considerations in the Design of Technology Assessments [7], and the RAND Corporation’s Disrupting Deterrence: Examining the Effects of Technologies on Strategic Deterrence in the 21st Century provide valuable insights but may compromise the objectivity and accuracy of their findings due to insufficient reliance on quantitative metrics [8]. On the other hand, certain studies overemphasize quantitative analysis, such as the KISTEP’s 2019 study using bibliometrics and patent analysis to evaluate South Korea’s technological gaps relative to the U.S., the European Union, Japan, and China [9], and the 2022 joint research by Alibaba Research Institute and Zhipu AI, which analyzed global digital technology trends through academic publications, patent filings, and innovation activity levels [10]. While these approaches offer precise data-driven insights, they often fail to incorporate expert judgment, which may lead to algorithmic bias. This gap leaves the field without a unified framework that can balance qualitative expert knowledge and quantitative data rigor, a shortfall that hinders both academic progress in STSST assessment methodologies and practical support for policymakers seeking evidence-based insights into national space technology strength.

**Figure 1:**
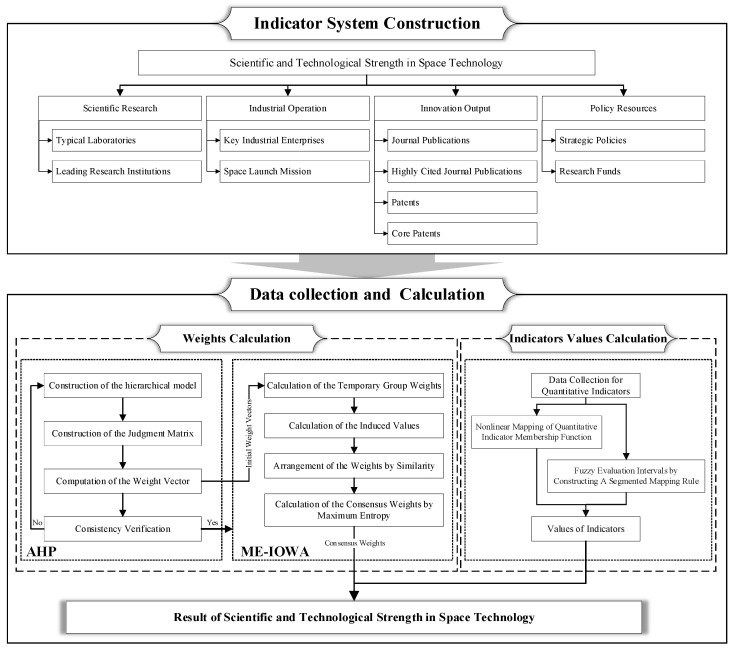
The research framework of the study.

This study aims to develop an innovative assessment methodology that integrates the Analytic Hierarchy Process (AHP) model, Induced Ordered Weighted Average (IOWA) operator, Maximum Entropy (ME) method, and a quantified indicator system to systematically evaluate and monitor national STSST, as shown in Figure 1. Methodologically, this framework delivers three critical advances: first, its benchmark indicators (covering four sub-dimensions: scientific research, industrial operation, innovation output, policy resources, and 10 specific quantitative indicators) resolve the over-subjectivity of qualitative-only assessments by embedding data-driven precision. Second, the methodology transforms experts’ qualitative evaluations of indicator importance into initial individual weight vectors via AHP—then uses the IOWA operator to aggregate these weights, with cosine similarity (between each expert’s vector and the group mean) as the induced value for reordering. This step uniquely mitigates outlier expert judgments and ensures weight assignments reflect collective, reliable insights. Second, the methodology transforms experts’ qualitative evaluations of indicator importance into initial individual weight vectors via AHP, then uses the IOWA operator to aggregate these weights, with cosine similarity as the induced value for reordering. This step uniquely mitigates outlier expert judgments and ensures weight assignments reflect collective, reliable insights. Third, the integration of the ME method to determine position weights adds a layer of rigor missing in existing workflows: it optimizes weight distribution to balance consensus and diverse expert perspectives, further enhancing the robustness of final indicator weights. By integrating the AHP model, ME method, and IOWA operator into a unified analytical framework, the methodology balances the qualitative judgment of domain experts with data-driven rigor, which ensures the rationality of the evaluation outcomes to a certain extent.

This paper is structured as follows: Section 2 introduces the research methods to assess STSST, which includes three parts: construction of the evaluation system, calculation of indicator weights based on AHP-ME-IOWA, and determination of evaluation standards. Section 3 presents the case study of the U.S. Section 2 will specifically elaborate on the methodology adopted, while the specific implementation steps, operational details, and data application will be explained in detail in Section 3. Section 4 summarizes the study, discusses current limitations, and outlines future directions.

## 2. Research Methods

This section is dedicated to explaining the theoretical framework and methodological process of the evaluation of STSST in our study. The process of practice for expert scoring, data collection, and calculation of the assessment will be set out in detail in Section 3.

### 2.1. Construction of the Evaluation System

The construction principle of the quantitative evaluation indicator system for STSST: When establishing the evaluation indicator system, the rationality of the indicators should be comprehensively considered, and the selection of indicators must be accurate and effective, following the principles of systematicity, simplicity, scientificity, and timeliness [11]. A thorough review of domestic and international literature on comprehensive research capacity assessments informed the process [2,3,4,5,6,7,8,9,10,12,13,14,15,16,17,18,19,20,21,22,23,24,25,26,27,28]. Additionally, we conduct adequacy assessments, redundancy analyses, and feasibility checks to identify necessity. Indicators are selected not only based on their contribution to STSST but also on their ability to effectively cover all aspects of STSST. Indicators are carefully considered.

Based on these foundations, a comprehensive evaluation indicator system was developed to operationalize STSST. Four sub-dimensions are defined at level 2: scientific research(*B*_1_), industrial operation(*B*_2_), innovation output(*B*_3_), and policy resources(*B*_4_). Inspired by RAND’s 2022 quantum-technology benchmarking framework, which compares China and the U.S. across four metric categories (research metrics, government activity metrics, private industry metrics, and technical metrics) [12]. This study adopts the same conceptual logic while tailoring the dimensions to space technology. These are further subdivided into 10 indicators, numbered accordingly in Table 1.

Typical Laboratories (*C*_1_): The basic value is the number of Typical Laboratories. Laboratories that are representative and advanced, reflecting the level of research infrastructure in the field of space science.

Leading Research Institutions (*C*_2_): The basic value is the number of the top 50 institutions in terms of publication volume in the WoS database. Research institutions that hold a leading position in the field of space science represent the top research level in this area.

Key Industrial Enterprises (*C*_3_): The basic value is the number of Key Industrial Enterprises. Key industrial enterprises that directly affect the integrity and maturity of the space industry chain, evaluated based on their distribution and influence in the space technology field.

Space Launch Mission (*C*_4_): The basic value is the number of successful launches per year. The frequency of space launch missions reflects a country’s activity and capability in space technology applications and space activities.

Journal Publications (*C*_5_): The basic value is the number of journal publications published according to WoS data, reflecting a country’s academic contribution and research activity in the field of space science.

Highly Cited Journal Publications (*C*_6_): The basic value is the number of highly cited papers from ESI core papers. The number of highly cited journal publications is an important criterion for measuring the international impact of research results.

Patents (*C*_7_): The basic value is the number of patent records from the DII. Reflecting the technological innovation capability and level of intellectual property protection in this field.

Core Patents (*C*_8_): The basic value is the number of patents identified by Price’s Law. Reflecting the core technological innovation capability and the depth and breadth of scientific research in this field.

Strategic Policies (*C*_9_): The basic value is the number of the Strategic policies. The government’s emphasis on decision-making in space science development reflects the strategic deployment and policy support of a country or region for space science.

Research Funds (*C*_10_): The basic value is the annual total national funding amount. The national total investment intensity in space science reflects a country’s financial support and investment intensity in this field.

In summary, these indicators together constitute a comprehensive and detailed quantitative evaluation system, which is crucial for STSST. The calculation method and data sources for each indicator value will be discussed in detail in Section 2.3 and Section 3.1.

### 2.2. Calculation of Indicator Weights

The calculation of indicator weights is a core link in the multi-indicator evaluation system, as the weight coefficient directly reflects the relative importance of each indicator in the evaluation context. Indicators with higher importance should correspond to larger weight coefficients, which is crucial for ensuring the scientificity and reliability of the final evaluation results. To accurately determine the indicator weights, this section integrates two key methods with complementary advantages. First, the AHP model is adopted to convert a single expert’s qualitative assessments of indicator importance into quantitative weight coefficients of that expert. Second, considering the complexity of practical multi-indicator evaluation problems and the differences in expert backgrounds, the ME-IOWA method is introduced for consensus adjustment of experts. This combination not only realizes the integration of multi-expert opinions but also automatically weakens the impact of outlier judgments, thereby improving the robustness of the final consensus weights.

#### 2.2.1. AHP-Based Expert Weighting

The weight reflects the importance of a factor or indicator in a given context [29]. In this work, we employed the AHP model to determine the weights of each indicator through expert qualitative assessments. The AHP model was constructed by Thomas L. Saaty in the early 1980s [30], which has a significant role in all segments of life [31,32,33]. The AHP model assigns weights to indicators using empirical data derived from pairwise comparisons of expert judgments, minimizing bias and including a consistency check.

Step 1: Construction of the hierarchical model

Based on the AHP model, the STSST is designated as the target layer *A*. Scientific research, industrial operation, innovation output, and policy resources are designated as the criteria layer *B*. All indicators, including typical laboratories, leading research institutions, key industrial enterprises, such as journal publications, highly cited journal publications, patents, and core patents, are designated as the indicator layer *C*. This structure is used to construct a hierarchical model for the quantitative evaluation indicator system of STSST.

Step 2: Construction of the judgment matrix

The judgment matrix is a comparison of the relative importance of all factors in the current layer compared to a factor in the previous layer. According to the established hierarchical structure model, the judgment matrix for *A*-*B_i_* (*i* = 1, 2, 3, 4) has been constructed. The relative importance ratings among the sub-dimensions were ascertained through a combination of questionnaire surveys and expert consultations, utilizing pairwise comparisons based on the 1–9 scale rating method. *b_ij_* represents the quantitative value of the degree to which indicator *B_i_* is more important than indicator *B_j_* (*j* = 1, 2, 3, 4) as shown in Table 2.

The judgment matrix for *A*-*B_i_* presents the criterion-layer judgment matrix for the target layer A, which is used to quantify the relative importance of each factor in the criterion layer (Scientific Research *B*_1_, Industrial Operation *B*_2_, Innovation Output *B*_3_, Policy Resources *B*_4_) with respect to the target layer STSST. The rows and columns of this matrix are all the four criterion-layer factors as shown in Table 3. Specifically:

1. The diagonal elements (e.g., *b*_11_ = 1, *b*_22_ = 1, etc.) indicate that a factor is equally important to itself, complying with the basic reciprocity rule of the judgment matrix;
2. The off-diagonal elements (e.g., *b*_12_ = 1/*b*_21_, etc.) reflect the quantitative comparison of importance between the row factor and the column factor.

This matrix serves as a key quantitative basis for subsequently calculating the weight of each criterion-layer factor.

Similarly, the indicator layer judgment matrix *B*_1_-*C_i_* (*i* = 1, 2), *B*_2_-*C_i_* (*i* = 3, 4), *B*_3_-*C_i_* (*i* = 5, 6, 7, 8), and *B*_4_-*C_i_* (*i* = 9, 10) can be derived.

Step 3: Computation of the weight vector

Based on the constructed judgment matrix *A*-*B_i_*, calculate the maximum eigenvalue *λ_max_* and its corresponding eigenvector *W_A_* = (*ω_A_*_1_, *ω_A_*_2_, *ω_A_*_3_, *ω_A_*_4_)^T^. In this step, each column vector of the judgment matrix is normalized. Subsequently, these normalized column vectors are summed on a row-by-row basis. Finally, the resultant row sums are normalized once more to yield an approximate eigenvector, which is the weight vector of the judgment matrix. The eigenvector is *W_A_* = (*ω_A_*_1_, *ω_A_*_2_, *ω_A_*_3_, *ω_A_*_4_)^T^, where *ω_Ai_* represents the weight of criterion *B_i_* for the overall target of a quantitative evaluation indicator system for STSST *A*. For the judgment matrix *A*-*B_i_* (*i* = 1, 2, 3, 4), the maximum eigenvalue *λ_A_* is calculated using mathematical methods:

(1) $$\lambda_{A}=\frac{1}{n}\sum_{i=1}^{n}\frac{{\left(A-B_{i}\cdot \omega_{A}\right)}_{i}}{\omega_{Ai}}$$

Similarly, for the judgment matrix *B*_1_-*C_i_* (*i* = 1, 2), *B*_2_-*C_i_* (*i* = 3, 4), *B*_3_-*C_i_* (*i* = 5, 6, 7, 8) and *B*_4_-*C_i_* (*i* = 9, 10), the maximum eigenvalue *λ_B_*_1_, *λ_B_*_2_, *λ_B_*_3_ and *λ_B_*_4_ is calculated using mathematical methods.

Step 4: Consistency verification

In the AHP progress, a consistent matrix represents an ideal state of a judgment matrix. It reflects the complete rationality and consistency of the decision-maker’s judgments. In practical applications, constructing a fully consistent judgment matrix is generally impractical, due to the complexity of real-world scenarios and the inherent uncertainty in human judgments. Therefore, the consistency degree of judgments is evaluated by comparing the differences between the actual judgment matrix and the consistent matrix, so as to ensure that the judgment matrix remains within an acceptable range.

**Theorem** **1.** *If matrix A is a consistent matrix, its maximum eigenvalue λ_max_ = n, where n denotes the order of matrix A. All other eigenvalues of A are 0.*

**Theorem** **2.** *An n-order positive reciprocal matrix is a consistent matrix if and only if its maximum eigenvalue λ_max_ = n. Moreover, if the positive reciprocal matrix is inconsistent, its maximum eigenvalue must satisfy λ_max_ > n.*

Based on the above theorems, after obtaining the calculated *λ_max_*, the value of the consistency indicator *CI* is defined as:

(2) $$CI=\frac{\lambda_{max}-n}{n-1}$$

When *CI* equals 0, it indicates perfect consistency, while a larger *CI* value suggests greater inconsistency. During this process, we utilize the random consistency indicator *RI*, as shown in Table 4, developed by Thomas L. Saaty. The construction method of the RI is to randomly construct 1000 positive reciprocal matrices and calculate the average value of the consistency indicator.

The ratio of *CI* to the *RI* is used as the criterion for assessing consistency, specifically:

(3) $$CR=\frac{CI}{RI}$$

The *CR*, or consistency ratio, is used to evaluate the consistency of the judgment matrix. If the *CR* value is less than 0.1, the judgment matrix is considered to have passed the consistency verification. However, if the *CR* value is 0.1 or higher, the judgment matrix fails the consistency verification and must be revised to improve its consistency. Therefore, for the judgment matrix *A*-*B_i_* (*i* = 1, 2, 3, 4), if the consistency ratio *CR_A_* is less than 0.1, the consistency verification is considered successful. It is the same for the judgment matrix *B*_1_-*C_i_* (*i* = 1, 2), *B*_2_-*C_i_* (*i* = 3, 4), *B*_3_-*C_i_* (*i* = 5,6,7,8) and *B*_4_-*C_i_* (*i* = 9, 10). If the consistency verification is successful, the corresponding weights can be confirmed.

#### 2.2.2. ME-IOWA Consensus Adjustment

With the continuous increase in the complexity of practical multi-indicator evaluation problems, involving only one expert in the evaluation process will affect the accuracy of the final weights. Moreover, experts have different focuses in their background experience and research directions, so multiple opinions can be integrated. Therefore, in the evaluation process, a team composed of multiple experts is usually formed to evaluate decision-making problems. This method introduces the IOWA method [34], which was proposed by Ronald R. Yager and is an extended form of the Ordered Weighted Averaging (OWA) operator. First, the cosine similarity between each expert’s weight vector and the group mean is used as the induced value, and the weights are reordered according to the level of similarity; then, the position weights are determined by the ME method, and the reordered weights are aggregated. Thus, while integrating multiple judgments, it automatically weakens the impact of outliers and improves the robustness of the consensus weights.

Step 1: Calculation of the temporary group weights

The temporary group weight vector is calculated by aggregating the individual weight vectors provided by all experts. Specifically, assuming there are *n* experts involved in the evaluation process, and each expert *k* (where *k* = 1, 2, …, *n*) provides a weight vector *W_k_* = (*ω_k__,_*_1_, *ω_k__,_*_2_, …, *ω_n,m_*)^T^ corresponding to *m* evaluation indicators, the temporary group weight vector $\bar{W}$ is determined by computing the arithmetic mean of the individual weight vectors across the expert dimension, expressed as:

(4) $$\bar{\omega_{i}}=\frac{1}{n}\sum_{k=1}^{n}\omega_{k,i}\;\left(i=1,2,\ldots ,m\right)$$

(5) $$\bar{W}={\left(\bar{\omega_{1}},\bar{\omega_{2},}\ldots ,\bar{\omega_{m}}\right)}^{Τ}$$

This temporary group weight serves as an initial reference for subsequent consensus adjustment, reflecting the aggregated tendency of multi-expert judgments on indicator importance.

Step 2: Calculation of the induced values

In our method, the cosine similarity *s* is introduced to measure the similarity between each expert’s weight vector and the temporary group weight vector. The cosine similarity is a widely used metric in vector space models, where a value closer to 1 indicates a higher degree of similarity between two vectors. The cosine similarity is selected to quantify the “distance” between individual expert vectors and the mean vector because it focuses on directional alignment rather than magnitude differences of vectors. This characteristic is particularly suitable for expert weight consensus measurement, as the core of weight assignment lies in the relative importance order of indicators (direction) rather than the absolute weight values (magnitude). The application of this approach is supported by existing literature. For instance, Ren et al. integrated cosine similarity into the normal cloud multi criteria group decision-making problem [35], and calculated the consensus degree of the group through cosine similarity to determine the degree of consensus among experts’ decision-making opinions. Mathematically, the cosine similarity *s_k_* for the *k*-th expert’s weight vector *W_k_* and the temporary group weight vector $\bar{W}$ is defined as:

(6) $$s_{k}=\cos\left(W_{k},\bar{W}\right)=\frac{W_{k}\cdot \bar{W}}{\left\|W_{k}\right\|\cdot \left\|\bar{W}\right\|}$$

Here, $\left\|W_{k}\right\|$ and $\left\|\bar{W}\right\|$ represent the Euclidean norm (L_2_ norm) of vectors $W_{k}$ and $\bar{W}$ respectively. The calculation method is the square root of the sum of the squares of each element in the n-dimensional vector.

By calculating the cosine similarity *s* for each expert’s weight vector, we obtain a set of induced values that reflect the degree of alignment between individual expert judgments and the initial group consensus. These induced values will then be used in the subsequent step to reorder the expert weight vectors, which is a crucial part of the consensus adjustment process in the IOWA method.

Step 3: Arrangement of the weights by similarity

The expert weight vectors are reordered based on the cosine similarity values {s_1_, s_2_, …, s*_n_*} calculated in Step 2. The core idea is that the higher the similarity between an expert’s weight vector and the temporary group weight vector, the further forward the position of the corresponding weight vector will be in the reordered sequence. This is because, in the subsequent OWA operation, positions closer to the front are associated with larger weight coefficients, meaning that expert judgments with higher similarity (and thus higher consistency with the group consensus) will have a greater influence on the final consensus weight.

Specifically, the reordering process is implemented as follows:

First, the expert weight vectors are sorted in descending order of their corresponding cosine similarity values *s*. That is, the weight vector with the highest *s* is placed at the first position *p*_1_, the one with the second-highest *s* is placed at *p*_2_, and so on, until the weight vector with the lowest *s* is placed at *p_n_*.

In cases where there is a tie in similarity values (for example, if the similarity values of expert 1 and expert 6 are both 0.9000), the order of these tied expert weight vectors can be random. In this specific context, to ensure a deterministic process, the tied weight vectors are ordered according to the order in which the experts were originally listed (i.e., the order of appearance in the expert group).

Through this reordering, the weight vectors are arranged in a sequence where those reflecting judgments more consistent with the group consensus are prioritized. This sequence {*p*_1_, *p*_2_, …, *p_n_*} will then be used in the next step to calculate the final consensus weight, leveraging the ordered structure to appropriately weight the expert judgments.

Step 4: Calculation of the consensus weights by ME

In this step, the rank-based ME method is adopted to determine the position weight *W_p_* = (*ω_p_*_1_, *ω_p_*_2_, …, *ω_pn_*)^T^, which are further used to aggregate the reordered expert weight vectors {*p*_1_, *p*_2_, …, *p_n_*} and obtain the final consensus weight vector. The core logic of this method is to assign larger position weights to the expert weight vectors that are more consistent with the group consensus (i.e., ranked earlier in Step 3), and the weight values decrease in reverse order of the ranking—this design ensures that judgments with higher similarity to the group opinion have a greater influence on the final consensus result, while still incorporating the information of all expert judgments.

The degree of *orness* associated with the *W_p_* is defined as:

(7) $$orness\left(W_{p}\right)=\frac{1}{n-1}\sum_{i=1}^{n}\left(n-i\right)\omega_{pi}$$

where *orness*(*W_p_*) = *α* ∈ [0, 1] is a measurement introduced by *Yager*, which can also be interpreted as the mode of decision-making in the aggregation process for weighting vector. The second characterizing measurement introduced by *Yager* is a measure of dispersion of the aggregation. The dispersion of *W_p_* is defined as:

(8) $$dispersion\left(W_{p}\right)=-\sum_{i-1}^{n}\omega_{pi}\ln\omega_{pi}$$

The principle of ME is integrated with IOWA operators to obtain the *W_p_*, which is designed to attain ME under a preset level of *orness*. The mathematical programming approach is described as:

(9) $$Maximize-\sum_{i-1}^{n}\omega_{pi}\ln\omega_{pi}$$

(10) $$Subject\;to:\frac{1}{n-1}\sum_{i=1}^{n}\left(n-i\right)\omega_{pi}=\alpha ,\;0\leq \alpha \leq 1$$

(11) $$\sum_{i=1}^{n}\omega_{pi}=1,\;0\leq \omega_{pi}\leq 1$$

The Lagrange multiplier method is applied to the IOWA operator equation to derive a polynomial equation, which is the key tool for determining the *W_p_* that satisfies the maximal entropy criterion. *W_p_* can be obtained by:

(12) $$\omega_{pi}=\sqrt[{n-1}]{\omega_{p1}^{n-i}\omega_{pn}^{i-1}}$$

and:

(13) $$\omega_{pn}=\frac{\left(\left(n-1\right)\alpha -n\right)\omega_{p1}+1}{\left(n-1\right)\alpha +1-n\omega_{p1}}$$

then:

(14) $$\omega_{p1}{\left[\left(n-1\right)\alpha +1-n\omega_{p1}\right]}^{n}={\left(\left(n-1\right)\alpha \right)}^{n-1}\left[\left(\left(n-1\right)\alpha -n\right)n\omega_{p1}+1\right]$$

Under this approach, the position weight *W_p_* is calculated by assigning different values to the *orness* parameter (*α* = 0.5, 0.6, 0.7,0.8, 0.9, 1.0). Table 5 illustrates the position weights under maximal entropy when *n* is 10.

When α is 0.5, the position weights tend to be more evenly distributed, which treats each expert’s input with roughly equal importance, weakening the distinction between higher-ranked and lower-ranked experts. As α increases, the weight for the top-ranked position (held by the expert most consistent with the group) becomes dominant, signifying that the scoring of this top-ranked expert carries much greater weight, while the inputs of lower-ranked experts are largely sidelined. The experts with consistent judgments will be prioritized to ensure the reliability of weights, while also retaining non-negligible weights for less consistent experts to avoid missing valuable niche insights [36].

### 2.3. Determination of Evaluation Standards

In the evaluation process of the indicator, different membership functions can lead to different results, which affect the credibility of the evaluation [37]. This study proposes a framework for constructing membership functions, tailored to the distribution characteristics of space technology indicators. Notably, indicators such as leading research institutions, space launch mission, journal publications, highly cited journal publications, patents, and core patents exhibit a significant right-skewed global distribution, with a small number of space-faring nations accounting for the majority of the top values. Under such distributions, traditional linear membership functions tend to cause “score saturation” for high-value indicators and “discrimination loss” for medium-to-low values. To address this, a power exponential membership function is adopted inspired by the fuzzy comprehensive evaluation method, leveraging its nonlinear mapping to preserve discriminability across all value ranges: it amplifies subtle differences among top performers while preventing undue compression of lower values, ensuring the evaluation remains objective, robust, and reflective of the actual global space technology hierarchy. The value *f*(*i*) of indicator i can be represented as:

(15) $$f\left(i\right)={\left(\frac{a}{t}\right)}^{k}\times 10$$

Here, for each indicator item, *a* represents the actual value of the evaluated country, *t* represents the total global actual value, and *k* is the exponent of the membership function. The theoretical basis of this formula is derived from the power-law transformation method of probability distribution [38], which can flexibly control the convexity and growth rate of the membership function by adjusting the parameter *k*. After experimental verification, when k = 0.2 (the experimentally verified optimal value), the function exhibits concave growth, which can effectively compress the marginal effects of high-value indicators and make the evaluation results more realistic.

For other indicators, a scoring standard was established by integrating quantitative data with expert judgment, as shown in Table 6. This standard converts precise values into fuzzy evaluation intervals by constructing a segmented mapping rule for indicator membership degrees.

Guided by Table 6, each indicator is scored and evaluated by industry experts, and then the evaluation results *f*(*i*) of indicator *i* can be represented as:

(16) $$f\left(i\right)={f\left(i\right)}_{1}\times \omega_{1}+{f\left(i\right)}_{2}\times \omega_{2}+\ldots +{f(i)}_{n}\times \omega_{n}$$

Here, *f*(*i*)*_n_* represents the score given by an expert for the indicator, and *ω_n_* represents the weight of that expert. In this study, experts were assigned equal weights during the scoring process for these indicators.

## 3. Case Study

As a traditional powerhouse in the space technology, the U.S. is selected as a practical case to validate the proposed method, through the evaluation of its STSST. There are three key reasons: (1) It is a long-standing global leader in space technology, with mature research infrastructure, a robust industrial chain, and sustained policy support, providing sufficient and high-quality data for empirical validation; (2) Its STSST has been widely discussed in existing literature, enabling cross-method comparisons to verify our model’s validity; (3) Insights from evaluating the U.S. can serve as a benchmark for other countries’ space technology development, enhancing the study’s practical value.

### 3.1. Data Description

To ensure the quality and accuracy of this work, the data from journal publications and patents used for this method were collected from the WoS database and the DII patent database. The WoS Core Collection is currently recognized as an authoritative citation indicator database, while the DII patent database is acknowledged worldwide for its high authority in patent literature. Based on a thorough investigation of the current status of scientific research and knowledge application in space technology [39,40,41], we determined the search strategy for literature and patents. The data from the past decade is more current and representative, providing a better reflection of the current academic field’s development trends and hotspots [42]. Considering the latency of paper publication and patent authorization, the relevant journal publications and patents published within the time range set to be from 1 January 2014, to 31 December 2023, were selected as the object of analysis. The retrieval was conducted on 8 January 2025.

Typical laboratories: based on available information, 17 well-known typical laboratories can be identified in the U.S., which are primarily categorized into three types: nationally funded laboratories, such as the Air Force Research Laboratory under the Department of Defense; NASA laboratories, such as the Ames Research Center and the Armstrong Flight Research Center; and university laboratories, such as the Lincoln Laboratory at the Massachusetts Institute of Technology.

Leading research institutions: the top 50 institutions in terms of publication volume in the WoS database can be considered as research leaders in the field. This perspective is not solely based on the number of papers published but also on their profound influence in academia and industry and their contributions to technological advancements. When analyzing the top 50 institutions by publication count retrieved from the WoS, it becomes clear that the U.S. enjoys a significant numerical advantage in space technology, with 25 institutions making it into the top 50. These institutions encompass national-level space research organizations like NASA, leading universities, and research divisions of private enterprises.

Key industrial enterprises: As the only enterprise consortium of the U.S. Department of Defense in space technology, the enterprises covered by the Space Enterprise Consortium (SpEC) can be used for analyzing the strength of key industrial enterprises. The U.S. Space Force relies on the SpEC to accelerate the research and development process of space technologies and equipment [43]. As of now, the SpEC has more than 750 members, focusing on the development of space technology [44]. Among them, there are not only well-known large-scale space enterprises in the U.S., such as SpaceX, Boeing, and so on, but also a large number of early-stage innovative and start-up commercial space enterprises and academic research institutions.

Space launch mission: In 2024, there were a total of 259 orbital launch attempts, marking a 17% increase from the previous record of 221 attempts in 2023, according to open-source data. This figure excludes suborbital launches, such as the four test flights of SpaceX’s Starship/Super Heavy and the two suborbital launches of Rocket Lab’s Electron HASTE variant. The U.S. accounted for 153 of these successful orbital launches. Globally, there were 251 successful orbital launches in total [45].

Journal publications: According to WoS data, overall, a total of 54,406 papers were published worldwide over the past decade, with 16,608 published in the U.S., ranking first globally. The U.S. has demonstrated high levels of activity and productivity in academic output within space technology. From 1256 papers in 2014 to 1789 papers in 2023, the U.S. has shown a fluctuating but overall upward trend in paper publication numbers. The performance of the U.S. becomes even more prominent when considering key metrics such as average citations per paper (28.24) and H-index (203).

Highly cited journal publications: This indicator analyzes the highly cited paper data from ESI core papers in space technology as the research basis. Through manual cleaning and deduplication, a total of 594 highly cited papers were generated from 2014 to 2023, of which 314 were highly cited papers by the U.S. researchers.

Patents: The number of patents is a crucial indicator of a country’s technological innovation capability and industrial competitiveness. According to data from the DII, there are 142,140 records worldwide between 2014 and 2023. The U.S. has a total of 31,602 patent records in space technology, with 55% representing new patent applications and 45% successfully granted, which likely reflects the higher efficiency and superior quality of the U.S. space technology research and development. The number of patents in the U.S. is not far ahead globally, which may be due to the limitations of the national defense intellectual property confidentiality management system on patent applications of American space technology companies.

Core patents: Typically, if a patent document is frequently cited, it indicates that the patent has a significant influence on subsequent research and is likely to be a foundational or core patent in the field. This work employs Price’s Law to identify core patents. Price’s Law states that within a particular subject area, half of the papers are written by a group of highly productive authors, and the number of authors in this group is approximately equal to the square root of the total number of authors. In the context of patent research, we can draw an analogy between authors and patents based on their shared adherence to the law of “a few core elements dominating overall contributions”. Specifically, the role of “number of papers” in measuring an author’s academic contribution in the academic field is equivalent to the role of “citation frequency” in evaluating a patent’s technical influence in the patent field. This analogy enables the rapid identification of core elements through quantitative thresholds, significantly reducing the cost of identifying core patented technologies. Defining *N_max_* as the maximum citation frequency, in the DII, the maximum citation frequency retrieved is 1759. By substituting this value into Price’s Law [46], the threshold for the number of core patent citations *M* can be expressed as:

(17) $$M=0.749\sqrt{N_{max}}$$

As a result, patents with a citation frequency of 31 or more are considered core patents. In the DII, a total of 3676 core patents were identified, with the U.S. institutions contributing 3280.

Strategic policies: The space strategy of the U.S. is an important component of its overall national security strategy and global strategy, which has a significant impact and significance in promoting the sustainable development of the space industry. The strategic thinking of the US space industry began in the 1950s. At different historical stages, successive US governments have continuously revised and improved their space strategies and policies based on changes in global politics, economy, military, and other factors during their tenure, providing top-level guidance and implementation guidance for the development of the space industry at the national level, and clarifying future development directions and priorities. As of 2024, there are over 145 relevant space strategies.

Research funds: The US Fiscal Year 2024 Defense Authorization Act provides a total of $30.1 billion in funding for the Department of Defense’s Space Force [47]. In addition, NASA’s budget is $24.875 billion. The budget for OSC under NOAA is $65 million. The budget for the FAA Office of Commercial Space Transportation is $42 million. The total amount above is $55.082 billion [48].

To ensure that the data is suitable for our evaluation framework, we have applied specific standards to transform the raw data, as detailed in Section 2.3. This transformation process is crucial for ensuring the accuracy and reliability of our subsequent analysis. The transformed data is then used in Section 3.3 for the calculations and evaluations that form the basis of our results.

### 3.2. Indicator Weights Based on AHP-ME-IOWA

The selection of experts is crucial to obtain an accurate judgment matrix [49]. This study selected 10 experts from the China Aerospace Academy of Systems Science and Engineering to assign weights to STSST, all of whom have profound professional experience in space technology. The “Questionnaire on Indicators Weights of Scientific and Technological Strength in Space Technology” is distributed for this purpose, which is provided in the Supplementary Materials. Details of the experts are shown in Table 7. These experts have the following characteristics: (1) Working and researching in space technology for over 5 years; (2) Having rich experience or knowledge of assessment in space technology. The extensive research or work experience of the experts helps them to construct accurate judgment matrices. Each participant was asked to score various evaluation criteria using a 1-to-9 rating scale. The questionnaire included an importance scale table to guide respondents in providing consistent and rational pairwise comparisons across multiple criteria. In addition, a hierarchical structure of the STSST was provided to help experts accurately understand the analytical framework and evaluation logic. Ten questionnaires were distributed, and all were effectively recovered, achieving a 100% recovery rate, sufficient for analysis.

After aggregating all expert scores from the questionnaires, we strictly followed the procedure described in Section 2.2 to calculate the weights. First, ten pairwise comparison matrices were constructed from the valid questionnaires to derive each expert’s indicator weights. Then, the consistency test was conducted. All experts’ matrices’ *CR* values are below 0.1, satisfying the consistency requirement and ensuring the reliability of the subsequent calculations. Since the AHP calculation process is consistent across the 10 experts, only the complete AHP of one expert is presented herein to ensure conciseness while maintaining methodological transparency.

Table 8 presents the criteria layer judgment matrix for target *A*, which quantitatively reflects the pairwise comparison results of the importance among four aspects: scientific research *B*_1_, industrial operation *B*_2_, innovation output *B*_3_, and policy resources *B*_4_ in the STSST. With the maximum eigenvalue of 4.021, *CI* of 0.007, and *CR* of 0.008 (less than 0.1), the matrix passes the consistency test, indicating that the pairwise comparison results are reasonable and reliable.

Table 9, Table 10, Table 11 and Table 12 present the judgment matrices for scientific research *B*_1_, industrial operation *B*_2_, innovation output *B*_3_, and policy resources *B*_4_, along with their respective consistency verification results. All these judgment matrices have successfully passed the consistency test, as shown in the tables. This indicates that the pairwise comparison results within each matrix are logically consistent and reliable, thereby providing a solid foundation for subsequent analytical work.

Next, using the group-mean weight vector as the reference, the cosine similarity between each expert’s weight vector and this reference is computed. With the results rounded to 4 decimal places, the weight vectors of experts are reordered from high to low similarity. Through experiments, it is concluded that when α(*orness*) is set to 0.7, position weights meets the practical requirements better. It preserves the IOWA’s ranking logic by giving more weight to higher-ranked (more consistent) experts, while still retaining non-negligible weights for lower-ranked experts to avoid missing valuable insights, thus striking a balanced integration of multi-expert judgments. The final consensus weight is calculated with the induced ordered weights and the position weights determined by the ME method. All the data in this progress are listed in Table 13, Table 14, Table 15, Table 16 and Table 17.

**Figure 2:**
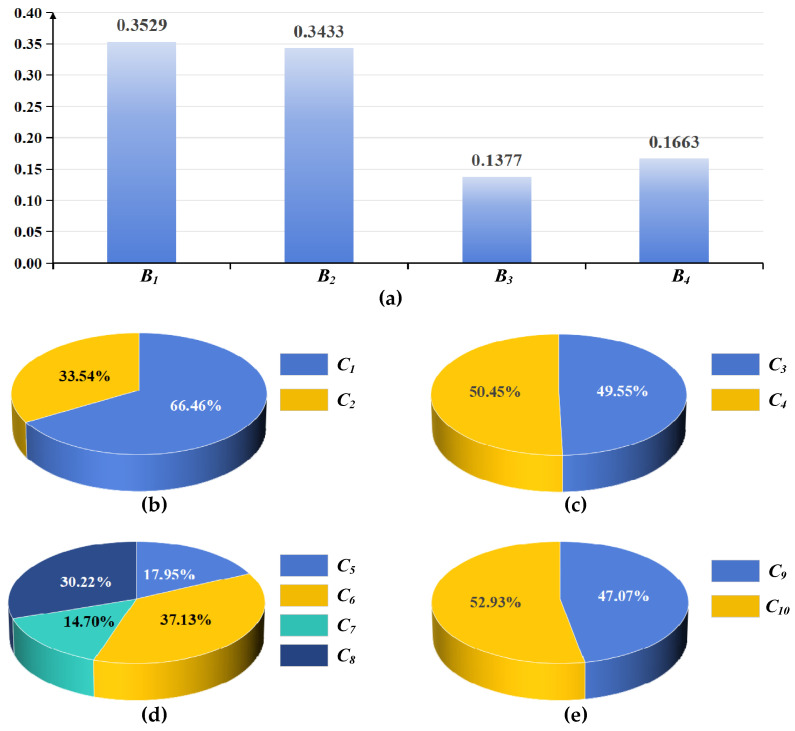
Consensus Weights calculated by AHP-ME-IOWA: (**a**) Weights of indicators in *A*; (**b**) Weights of indicators in *B*_1_; (**c**) Weights of indicators in *B*_2_; (**d**) Weights of indicators in *B*_3_; (**e**) Weights of indicators in *B*_4_.

Figure 2 presents the weight calculation results calculated by AHP-ME-IOWA for each indicator within the indicator system. These weights are indicative of the perceived importance of each indicator in the overall evaluation framework. In Figure 2a, the vertical axis quantifies the weight values, which range from 0 to 1, with higher values suggesting greater significance in the assessment process. The horizontal axis lists the sub-dimensions. In Figure 2b–e, the weight distribution of the indicators within each sub-dimension is presented in percentage form.

### 3.3. Indicator Values and Aggregation

Based on the membership function Equation (15), Table 6, and Equation (16), all of which are found in Section 2.3, the calculated values of each indicator are applied to the evaluation standard to obtain the corresponding calculation values for each indicator. For the indicators including leading research institutions, space launch missions, journal publications, highly cited journal publications, patents, and core patents, the calculated values are obtained by substituting U.S. data and global data into the membership function Equation (15); for the indicators such as typical laboratories, key industrial enterprises, strategic policies, and research funds, the calculated values are derived from the evaluation results given by 10 experts (the same as the experts who scored in the AHP) under the guidance of Table 6 and Equation (16). The indicator data were normalized to a scale of ten, with scores retained to two decimal places. These normalized scores were then multiplied by the weights of the respective evaluation indicators to calculate the final scores for each dimension using the weighted average method. The specific scores for the main dimension, sub-dimensions, and indicators are summarized in Table 18. Overall, the comprehensive scientific research capability value for the U.S. in space technology is 8.73.

### 3.4. Comparison and Discussion

On one hand, to verify the sensitivity of the AHP-ME-IOWA model to the core parameter orness (*α*), this study selected a total of 6 sets of decision preference coefficients, namely *α* = 0.5, 0.6, 0.7, 0.8, 0.9, and 1.0, covering the entire range from “fully averaged decision (*α* = 0.5)” to “extremely consensus-based decision (*α* = 1.0)”. Based on the position weights corresponding to each set of α values, the weights of the secondary dimensions, the weights of the tertiary indicators, and the comprehensive score in the assessment of the United States’ STSST were recalculated as shown in Table 19, to analyze the impact of *α* value changes on the assessment results.

The results of the sensitivity analysis show that under the 6 sets of *α* values, the comprehensive score of the United States’ STSST ranges from 8.7182 to 8.7377 (out of 10 points), with a range of only 0.0195 points. The weight ranking of the secondary dimensions remains stable without significant fluctuations, and the weight fluctuation range of the core tertiary indicators is all less than 3%, indicating that the model has extremely low sensitivity to changes in the *α* value. This stability is partly due to the fact that the 10 experts all come from the same institution and have similar cognitive backgrounds and evaluation logics in the space field, resulting in small differences in the initial weight vectors (with cosine similarity all greater than 0.9775). In addition, the evaluation indicators are all core explicit indicators of Scientific and Technological Strength in Space Technology (such as the number of typical laboratories and the proportion of core patents), and the importance of these indicators conforms to industry consensus. In addition, the ME-IOWA operator balances consensus and diversity through the maximum entropy principle, the power exponential membership function compresses the marginal differences in high-value indicators, and the absolute advantage of the United States in the space field forms a “stable basic framework”. All these factors together weaken the impact of *α* value changes on the results.

On the other hand, the STSST of the U.S., Russia, and Japan has been evaluated by our method as well as by method AHP-Delphi and method AHP-CIE. Method AHP-Delphi [14] integrates the AHP model with the Delphi method. It determines the weights of indicators through expert consultation and evaluation criteria through bibliometric analysis to assess STSST of various countries. Method AHP-CIE [50] employs the AHP and comprehensive indicator evaluation method, integrating qualitative methods based on expert experience with quantitative methods based on mathematical statistics, to quantify and assess STSST. In the method comparison experiment, the same indicator system proposed in Table 1, as well as the expert scores, were used by all three methods. The comparative results of the three methods for the three countries are shown in Table 20.

Table 20 indicates that our method has assigned scores of 8.73, 3.56, and 3.22 to the U.S., Russia, and Japan, respectively, which aligns with the actual global development situation [51], suggesting a certain degree of feasibility in our approach. The AHP-Delphi method has given scores of 8.96, 2.92, and 3.46 to the U.S., Russia, and Japan, respectively. The AHP-CIE method has assigned scores of 9.05, 4.10, and 3.58. The difference in ranking by the method AHP-Delphi may be due to its greater reliance on expert consultation, where subjective judgments can influence the final rankings. In all methods, the U.S. ranks first, while Russia and Japan have relatively close scores. The general trend of STSST generated by our AHP-ME-IOWA model is consistent with the global space technology strength landscape reported in existing literature [14,50], further validating the reliability of our results.

## 4. Conclusions

This paper presents an innovative model that combines the AHP model, IOWA operator, ME method, and a quantitative indicator system to systematically evaluate and continuously track STSST. The key strength of the model lies in its structured approach to weight determination: AHP first converts experts’ qualitative judgments on indicator importance into initial individual weight vectors, addressing the challenge of quantifying subjective professional insights that pure data-driven methods often overlook. The ME-IOWA operator then enhances this process by introducing two critical improvements. The cosine similarity is used between each expert’s weight vector and the temporary group mean as an induced value to reorder individual weights, ensuring that weights align with collective expert consensus while preserving diverse but rational perspectives. The ME method is employed to calculate position weights for the reordered experts’ weight vectors, a design that effectively weakens the distorting impact of outlier judgments under given constraints, thus enhancing the robustness of the final composite weights. In addition, by adopting internationally recognized cross-border comparison indicators, this method provides a dynamic indicator system that can adapt to the rapid development of space technology. The total research framework balances the objectivity of quantitative data with the depth of expert judgment. A systematic evaluation of the scientific research capabilities of the U.S. was conducted through this method, conducting an initial empirical validation of the proposed method.

The following discussion will explore the limitations of our method to provide a balanced perspective. Firstly, due to data lag—particularly for space technology patents and scientific publications sourced from the WoS and DII—the dataset may not fully capture the latest activities of emerging space-faring nations, whose rapid technological progress could be underestimated. Secondly, the 10 experts surveyed in this case study are all from the China Aerospace Academy of Systems Science and Engineering; while they hold rich professional knowledge in space technology, this single-institution selection may lead to homogeneous judgments on indicator weights, failing to incorporate diverse views from other sectors like universities or commercial space enterprises. Additionally, the use of a single aggregated value for each indicator overlooks the unique influences of specific entities within each indicator category, limiting the evaluation’s depth and granularity.

Future research could address these limitations by incorporating additional data sources and expanding the scope of experts and countries. To mitigate data lag, additional timely data sources will be integrated alongside traditional databases. The scope of expert selection will be expanded to include professionals from diverse backgrounds, such as researchers from universities, engineers from international space enterprises, and government policy analysts, to enhance the objectivity of weight assignments. Furthermore, recognizing the limitation of single aggregated indicator values, in-depth research will be conducted on individual entities within each indicator, to capture fine-grained impacts and improve the precision of STSST assessment.

## Figures and Tables

**Table 1:** Evaluation indicator system for STSST.

Main Dimension (Target Layer *A*)	Sub-Dimensions (Criteria Layer *B*)	Indicators (Indicator Layer *C*)	References
STSST(*A*)	Scientific Research (*B*_1_)	Typical Laboratories (*C*_1_)	[12,13]
		Leading Research Institutions (*C*_2_)	[14,15]
	Industrial Operation (*B*_2_)	Key Industrial Enterprises (*C*_3_)	[3,16]
		Space Launch Mission (*C*_4_)	[17,18]
	Innovation Output (*B*_3_)	Journal Publications (*C*_5_)	[6,19,20]
		Highly Cited Journal Publications (*C*_6_)	[4,10,21]
		Patents (*C*_7_)	[5,9,22,23]
		Core Patents (*C*_8_)	[24,25]
	Policy Resources (*B*_4_)	Strategic Policies (*C*_9_)	[17,26]
		Research Funds (*C*_10_)	[27,28]

**Table 2:** AHP model importance scale table.

Scale	Meaning
1	Indicates that two factors are of equal importance compared with each other
3	Indicates that the former is slightly more important than the latter
5	Indicates that the former is significantly more important than the latter
7	Indicates that the former is significantly more important than the latter
9	Indicates that the former is extremely more important than the latter
Backwards	If the ratio of the importance of factor *i* to that of factor *j* is *b_ij_*, then the ratio of the importance of factor *j* to factor *i* is *b_ji_* = 1/*b_ij_*
*b_ij_* = an even number between 1~9	The importance of the *i* element over the *j* element is between neighboring judgments.

**Table 3:** Regarding the criteria layer judgment matrix for the target *A*.

STSST *A*	Scientific Research *B*_1_	Industrial Operation *B*_2_	Innovation Output *B*_3_	Policy Resources *B*_4_
Scientific Research *B*_1_	*b*_11_ = 1	*b*_12_	*b*_13_	*b*_14_
Industrial Operation *B*_2_	*b*_21_ = 1/*b*_12_	*b*_22_ = 1	*b*_23_	*b*_24_
Innovation Output *B*_3_	*b*_31_ = 1/*b*_13_	*b*_32_ = 1/*b*_23_	*b*_33_ = 1	*b*_34_
Policy Resources *B*_4_	*b*_41_ = 1/*b*_14_	*b*_42_ = 1/*b*_24_	*b*_43_ = 1/*b*_34_	*b*_44_ = 1

**Table 4:** Table of *RI* values.

Size of Matrix (*n*)	1	2	3	4	5	6	7	8
*RI*	0	0	0.52	0.89	1.12	1.26	1.36	1.41

**Table 5:** The position weights under maximal entropy (*n* = 10).

Weight	*ω_p_*_1_	*ω_p_*_2_	*ω_p_*_3_	*ω_p_*_4_	*ω_p_*_5_	*ω_p_*_6_	*ω_p_*_7_	*ω_p_*_8_	*ω_p_*_9_	*ω_p_*_10_
*α* = 0.5	0.1000	0.1000	0.1000	0.1000	0.1000	0.1000	0.1000	0.1000	0.1000	0.1000
*α* = 0.6	0.1569	0.1404	0.1256	0.1123	0.1005	0.0899	0.0804	0.0720	0.0644	0.0576
*α* = 0.7	0.2336	0.1840	0.1450	0.1143	0.0901	0.0710	0.0559	0.0441	0.0347	0.0274
*α* = 0.8	0.3427	0.2272	0.1506	0.0998	0.0662	0.0439	0.0291	0.0193	0.0128	0.0085
*α* = 0.9	0.5250	0.2495	0.1186	0.0564	0.0268	0.0127	0.0061	0.0029	0.0014	0.0007
*α* = 1.0	1.0000	0.0000	0.0000	0.0000	0.0000	0.0000	0.0000	0.0000	0.0000	0.0000

**Table 6:** Indicator scoring table.

Indicator	1–4 Points	4–7 Points	7–10 Points
Typical Laboratories	<5	5–10	>10
Key Industrial Enterprises	<300	300–600	>600
Strategic Policies	<60	60–100	>100
Research Funds (Billion Dollars)	<5	5–20	>20

**Table 7:** Details of the experts.

Number	Professional Title	Work Years
Expert 1	Professor	18
Expert 2	Professor	15
Expert 3	Associate Professor	12
Expert 4	Associate Professor	10
Expert 5	Senior Engineer	17
Expert 6	Senior Engineer	12
Expert 7	Senior Engineer	10
Expert 8	Senior Engineer	9
Expert 9	Engineer	7
Expert 10	Engineer	5

**Table 8:** Regarding the criteria layer judgment matrix for the target *A* with the actual value.

STSST *A*	Scientific Research *B*_1_	Industrial Operation *B*_2_	Innovation Output *B*_3_	Policy Resources *B*_4_
Scientific Research *B*_1_	1.000	1.000	2.000	3.000
Industrial Operation *B*_2_	1.000	1.000	2.000	2.000
Innovation Output *B*_3_	0.500	0.500	1.000	1.000
Policy Resources *B*_4_	0.333	0.500	1.000	1.000
*λ_max_* = 4.021, *CI* = 0.007, *CR* = 0.008 < 0.1				

**Table 9:** Judgment matrix for scientific research *B*_1_ with the actual value.

Scientific Research *B*_1_	Typical Laboratories *C*_1_	Leading Research Institutions *C*_2_
Typical Laboratories *C*_1_	1.000	2.000
Leading Research Institutions *C*_2_	0.500	1.000
*λ_max_* = 2.000, *CI* = 0.000, *CR* = 0.000 < 0.1		

**Table 10:** Judgment matrix for industrial operation *B*_2_ with the actual value.

Industrial Operation *B*_2_	Key Industrial Enterprises *C*_3_	Space Launch Mission *C*_4_
Key Industrial Enterprises *C*_3_	1.000	1.000
Space Launch Mission *C*_4_	1.000	1.000
*λ_max_* = 2.000, *CI* = 0.000, *CR* = 0.000 < 0.1		

**Table 11:** Judgment matrix for innovation output *B*_3_ with the actual value.

Innovation Output *B*_3_	Journal Publications *C*_5_	Highly Cited Journal Publications *C*_6_	Patents *C*_7_	Core Patents *C*_8_
Journal Publications *C*_5_	1.000	0.500	1.000	1.000
Highly Cited Journal Publications *C*_6_	2.000	1.000	2.000	1.000
Patents *C*_7_	1.000	0.500	1.000	0.500
Core Patents *C*_8_	1.000	1.000	2.000	1.000
*λ_max_* = 4.061, *CI* = 0.020, *CR* = 0.023 < 0.1				

**Table 12:** Judgment matrix for policy resources *B*_4_ with the actual value.

Policy Resources *B*_4_	Strategic Policies *C*_9_	Research Funds *C*_10_
Strategic Policies *C*_9_	1.000	2.000
Research Funds *C*_10_	0.500	1.000
*λ_max_* = 2.000, *CI* = 0.000, *CR* = 0.000 < 0.1		

**Table 13:** Expert weights and consensus weight values of sub-dimensions.

Rank	Scientific Research *B*_1_	Industrial Operation *B*_2_	Innovation Output *B*_3_	Policy Resources *B*_4_	Induced Value	Position Weights
Expert 6	0.3536	0.3536	0.1317	0.1612	0.9993	0.2336
Expert 1	0.3620	0.3263	0.1632	0.1485	0.9972	0.1840
Expert 2	0.3298	0.3155	0.1524	0.2024	0.9965	0.1450
Expert 9	0.3472	0.3829	0.1276	0.1423	0.9949	0.1143
Expert 8	0.3655	0.3905	0.0971	0.1468	0.9926	0.0901
Expert 10	0.3954	0.3676	0.1145	0.1225	0.9919	0.0710
Expert 3	0.3625	0.2802	0.1197	0.2375	0.9868	0.0559
Expert 4	0.2881	0.3381	0.2048	0.1690	0.9840	0.0441
Expert 5	0.2817	0.3373	0.1409	0.2401	0.9832	0.0347
Expert 7	0.4527	0.2775	0.0990	0.1708	0.9775	0.0274
Consensus Weights	0.3529	0.3433	0.1377	0.1663		

**Table 14:** Expert weights and consensus weight values of *B*_1_ indicators.

Rank	Typical Laboratories *C*_1_	Leading Research Institutions *C*_2_	Induced Value	Position Weights
Expert 1	0.6667	0.3333	0.9995	0.2336
Expert 3	0.6667	0.3333	0.9995	0.1840
Expert 4	0.6667	0.3333	0.9995	0.1450
Expert 5	0.6667	0.3333	0.9995	0.1143
Expert 6	0.6667	0.3333	0.9995	0.0901
Expert 10	0.6667	0.3333	0.9995	0.0710
Expert 7	0.7500	0.2500	0.9852	0.0559
Expert 8	0.7500	0.2500	0.9852	0.0441
Expert 2	0.5000	0.5000	0.9578	0.0347
Expert 9	0.5000	0.5000	0.9578	0.0274
Consensus Weights	0.6647	0.3354		

**Table 15:** Expert weights and consensus weight values of *B*_2_ indicators.

Rank	Key Industrial Enterprises *C*_3_	Space Launch Mission *C*_4_	Induced Value	Position Weights
Expert 1	0.5000	0.5000	0.9994	0.2336
Expert 2	0.5000	0.5000	0.9994	0.1840
Expert 3	0.5000	0.5000	0.9994	0.1450
Expert 4	0.5000	0.5000	0.9994	0.1143
Expert 5	0.5000	0.5000	0.9994	0.0901
Expert 6	0.5000	0.5000	0.9994	0.0710
Expert 8	0.5000	0.5000	0.9994	0.0559
Expert 9	0.5000	0.5000	0.9994	0.0441
Expert 10	0.5000	0.5000	0.9994	0.0347
Expert 7	0.3333	0.6667	0.9587	0.0274
Consensus Weights	0.4955	0.5046		

**Table 16:** Expert weights and consensus weight values of *B*_3_ indicators.

Rank	Journal Publications *C*_5_	Highly Cited Journal Publications *C*_6_	Patents *C*_7_	Core Patents *C*_8_	Induced Value	Position Weights
Expert 10	0.1801	0.3973	0.1443	0.2783	0.9988	0.2336
Expert 6	0.1632	0.3620	0.1485	0.3263	0.9976	0.1840
Expert 7	0.1632	0.3620	0.1485	0.3263	0.9976	0.1450
Expert 4	0.1974	0.3678	0.1170	0.3178	0.9973	0.1143
Expert 1	0.2048	0.3381	0.1690	0.2881	0.9950	0.0901
Expert 9	0.2048	0.3381	0.1690	0.2881	0.9950	0.0710
Expert 2	0.1690	0.3381	0.2048	0.2881	0.9903	0.0559
Expert 3	0.2024	0.3155	0.1524	0.3298	0.9902	0.0441
Expert 5	0.1820	0.4736	0.0971	0.2473	0.9800	0.0347
Expert 8	0.1296	0.4959	0.0858	0.2887	0.9748	0.0274
Consensus Weights	0.1795	0.3713	0.1470	0.3022		

**Table 17:** Expert weights and consensus weight values of *B*_4_ indicators.

Rank	Strategic Policies *C*_9_	Research Funds *C*_10_	Induced Value	Position Weights
Expert 2	0.5000	0.5000	0.9889	0.2336
Expert 3	0.5000	0.5000	0.9889	0.1840
Expert 6	0.5000	0.5000	0.9889	0.1450
Expert 7	0.5000	0.5000	0.9889	0.1143
Expert 9	0.5000	0.5000	0.9889	0.0901
Expert 10	0.5000	0.5000	0.9889	0.0710
Expert 1	0.3333	0.6667	0.9851	0.0559
Expert 4	0.3333	0.6667	0.9851	0.0441
Expert 5	0.3333	0.6667	0.9851	0.0347
Expert 8	0.2500	0.7500	0.9509	0.0274
Consensus Weights	0.4707	0.5294		

**Table 18:** Values of the quantitative evaluation indicator system for STSST.

Main Dimension	Values	Sub-Dimensions	Values	Indicators	Values
STSST	8.73	Scientific Research	8.37	Typical Laboratories	8.20
				Leading Research Institutions	8.71
		Industrial Operation	8.83	Key Industrial Enterprises	8.60
				Space Launch Mission	9.06
		Innovation Output	8.72	Journal Publications	7.88
				Highly Cited Journal Publications	8.80
				Patents	7.40
				Core Patents	9.77
		Policy Resources	9.27	Strategic Policies	9.00
				Research Funds	9.50

**Table 19:** Weight values and STSST results under different *α*.

*Orness* (α)	STSST	*B*_1_	*B*_2_	*B*_3_	*B*_4_	*C*_1_	*C*_2_	*C*_3_	*C*_4_	*C*_5_	*C*_6_	*C*_7_	*C*_8_	*C*_9_	*C*_10_
0.5	8.7377	0.3539	0.3369	0.1351	0.1741	0.6500	0.3500	0.4833	0.5166	0.1796	0.3788	0.1436	0.2979	0.4250	0.5750
0.6	8.7313	0.3531	0.3408	0.1362	0.1699	0.6590	0.3410	0.4904	0.5096	0.1802	0.3733	0.1461	0.3005	0.4495	0.5505
0.7	8.7279	0.3529	0.3433	0.1377	0.1663	0.6647	0.3354	0.4955	0.5046	0.1795	0.3713	0.1470	0.3022	0.4707	0.5294
0.8	8.7245	0.3528	0.3444	0.1394	0.1634	0.6672	0.3329	0.4986	0.5015	0.1778	0.3731	0.1467	0.3025	0.4877	0.5124
0.9	8.7224	0.3532	0.3445	0.1408	0.1616	0.6671	0.3330	0.4999	0.5002	0.1758	0.3800	0.1456	0.2988	0.4981	0.5020
1.0	8.7182	0.3536	0.3536	0.1317	0.1612	0.6667	0.3333	0.5000	0.5000	0.1801	0.3973	0.1443	0.2783	0.5000	0.5000

**Table 20:** Scores and rankings of evaluation utility values.

Method	① The U.S.	② Russia	③ Japan	Ranking
Our proposed method, AHP-ME-IOWA	8.73	3.56	3.22	① > ② > ③
Method AHP-Delphi	8.96	2.92	3.46	① > ③ > ②
Method AHP-CIE	9.05	4.10	3.58	① > ② > ③

## Data Availability

The data are already in the graphs and references of the paper, and further inquiries can be made by contacting the corresponding author.

## Supplementary Materials

The following supporting information can be downloaded at: https://www.mdpi.com/article/10.3390/e27111141/s1, Questionnaire on Indicator Weights of National Space Technology Scientific and Technological Strength.

## Abbreviations

The following abbreviations are used in this manuscript:

STSST	Scientific and Technological Strength in Space Technology
AHP	Analytic Hierarchy Process
IOWA	Induced Ordered Weighted Average
ME	Maximum Entropy
WoS	Web of Science
DII	Derwent Innovations Index
OWA	Ordered Weighted Averaging
NASA	National Aeronautics and Space Administration
SpEC	Space Enterprise Consortium
OSC	Office of Space Commerce
NOAA	National Oceanic and Atmospheric Administration
FAA	Federal Aviation Administration
PCC	Pearson product–moment correlation coefficient
