# Supplementary material for: An AHP-ME-IOWA Model for Assessing National Space Technology Scientific and Technological Strength: A Case Study of the United States
Source: Entropy (Basel). 2025 Nov 6;27(11):1141. doi: 10.3390/e27111141 (PMC12651672; doi:10.3390/e27111141)
Supplement: Supplementary file 1 [file entropy-27-01141-s001.zip › entropy-3923039-supplementary.pdf]

Questionnaire ID：_______

# Questionnaire on Indicator Weights of National Space Technology Scientific and Technological Strength

Dear Sir/Madam,

To determine the weights of the evaluation indicator system for scientific and technological strength in space technology, we kindly request your valuable time to complete the following questionnaire. This survey is anonymous, and the results will be used solely for research purposes without any negative impact on you. Please compare the relative importance of the two indicators mentioned in the questionnaire: The survey uses a 1–9 scaling method—please fill in the corresponding scale value in the appropriate position. The meanings and descriptions of the numerical scales are as follows:

1–9 Scale Definitions:

- 1 = Equally important (Two indicators are of the same importance.)
- 3 = Slightly more important (One indicator is slightly more important than the other.)
- 5 = Obviously more important (One indicator is obviously more important than the other.)
- 7 = Strongly more important (One indicator is strongly more important than the other.)
- 9 = Extremely more important (One indicator is extremely more important than the other.)
- 2, 4, 6, 8 = Intermediate values between the above adjacent scales.

*Reciprocal: If you believe that one indicator is less important than the other, please use the reciprocal value (e.g., if you rate one indicator as 3 times more important than the other, rate the reverse comparison as 1/3).*

The indicator system is shown in the following figure:

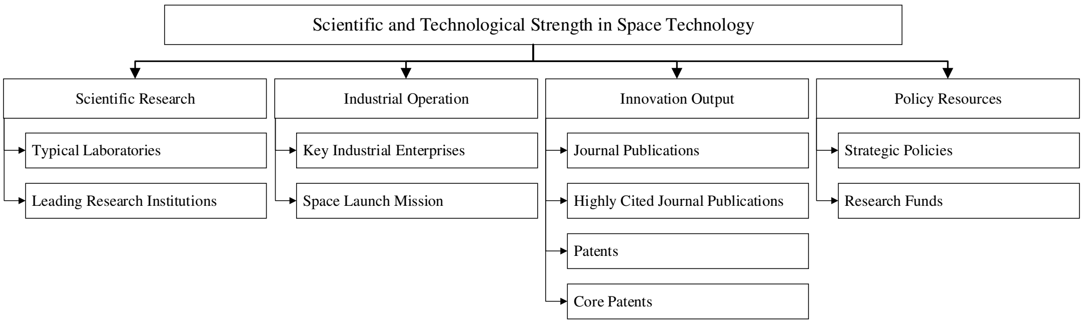


Main Dimension comparison:

| **Sub-Dimension** | **Importance Level** | **Sub-Dimension** |
|---|---|---|
| Scientific Research | | Industrial Operation |
| Scientific Research | | Innovation Output |
| Scientific Research | | Policy Resources |
| Industrial Operation | | Innovation Output |
| Industrial Operation | | Policy Resources |
| Innovation Output | | Policy Resources |

Sub-dimensions comparison:

1、"Scientific research" comparison

| **Indicators** | **Importance Level** | **Indicators** |
|---|---|---|
| Typical Laboratories | | Leading Research Institutions |

2、“Industrial operation” comparison

| **Indicators** | **Importance Level** | **Indicators** |
|---|---|---|
| Space Launch Mission | | Key Industrial Enterprises |

3、“Innovation output” comparison

| **Indicators** | **Importance Level** | **Indicators** |
|---|---|---|
| Journal Publications | | Highly Cited Journal Publications |
| Journal Publications | | Patents |
| Journal Publications | | Core Patents |
| Highly Cited Journal Publications | | Patents |
| Highly Cited Journal Publications | | Core Patents |
| Patents | | Core Patents |

4、“Policy resources” comparison

| **Indicators** | **Importance Level** | **Indicators** |
|---|---|---|
| Strategic Policies | | Research Funds |
